# Charting the importance of filamin A posttranslational modifications

**DOI:** 10.1042/BCJ20240121

**Published:** 2024-07-03

**Authors:** Kyle D. Shead, Veneta Salyahetdinova, George S. Baillie

**Affiliations:** School of Cardiovascular and Metabolic Health, University of Glasgow, Glasgow G128QQ, U.K.

**Keywords:** filamin A, phosphorylation, post translational modification, proteolysis

## Abstract

Filamin A is an essential protein in the cell cytoskeleton because of its actin binding properties and unique homodimer rod-shaped structure, which organises actin into three-dimensional orthogonal networks imperative to cell motility, spreading and adhesion. Filamin A is subject to extensive posttranslational modification (PTM) which serves to co-ordinate cellular architecture and to modulate its large protein-protein interaction network which is key to the protein's role as a cellular signalling hub. Characterised PTMs include phosphorylation, irreversible cleavage, ubiquitin mediated degradation, hydroxylation and O-GlcNAcylation, with preliminary evidence of tyrosylation, carbonylation and acetylation. Each modification and its relation to filamin A function will be described here. These modifications are often aberrantly applied in a range of diseases including, but not limited to, cancer, cardiovascular disease and neurological disease and we discuss the concept of target specific PTMs with novel therapeutic modalities. In summary, our review represents a topical ‘one-stop-shop’ that enables understanding of filamin A function in cell homeostasis and provides insight into how a variety of modifications add an extra level of Filamin A control.

## Introduction to filamins

**Figure 1.**
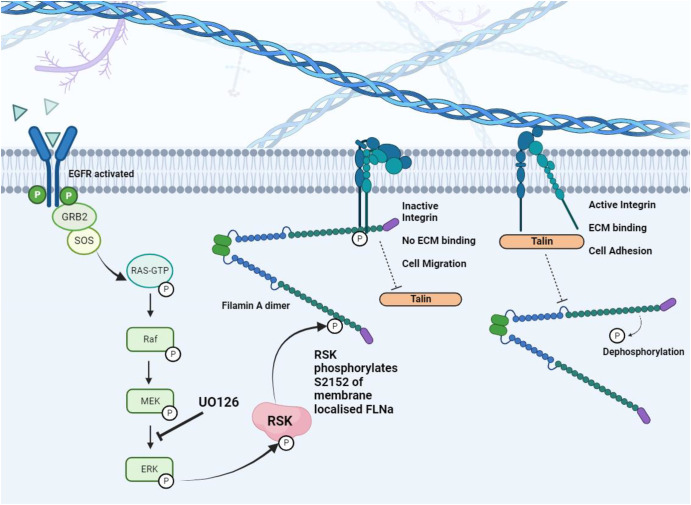
Filamin A phosphorylation by RSK in response to EGF-stimulation controls integrin activation dynamics and cell migratory status. P90 ribosomal S6 Kinase (RSK) is activated by epidermal growth factor receptor (EGFR) signalling after agonism by EGF. RAS-GTP mediated mitogen activated kinase (MAPK) signalling cascade activates RSK which translocates to the cell surface membrane pool of filamin A, phosphorylating S2152. This augments filamin A interaction with the fibronectin specific α_5_β_1_ integrin resulting in integrin inactivation by out-competing integrin activators such as Talin. Dephosphorylated filamin is out-competed by Talin to bind integrin forcing integrin activation and binding to fibronectin in the extracellular matrix. Figure made with BioRender.

The filamins are a group of actin binding, scaffold proteins consisting of three isoforms, Filamin A, B and C. Filamin A (previously termed actin binding protein-280, ABP-280) was the first isoform discovered after its isolation from rabbit alveolar macrophages, where it was shown to be a necessary cofactor in the activation of Mg^2+^ ATPase by F-actin [1]. Since its discovery many seminal studies (including discovery of the B and C filamin isoforms) characterised the main functions of filamins within the actin cytoskeleton, cytoplasmic contraction machinery, cell motility mechanisms, gelation and cell adhesion/spreading [2]. Filamin A and B isoforms are ubiquitously expressed whereas Filamin C expression is confined to smooth and skeletal muscle. This difference in intra-cellular segregation is reflected in the positions held by filamin isoforms where FLNA and B predominantly localise to actin stress fibres and cell cortex whereas FLNC localises to the sarcomeric Z-disc region [3,4]. The general structure of the filamins (depicted in Figure 1) is a distinct V-shaped homodimer where each monomer contains an N-terminal actin binding domain (consisting of two calponin-homology domains), followed by 24-IgG like repeats forming two rod-domains (IgG1-15 and IgG16-24) interspaced by two flexible hinge regions. The final IgG24 repeat acts as a dimerisation interface to form the dimer at the C-terminus. Isoform differences in structure are germane to function as FLNA for instance, contains a transmembrane domain in its N-terminus allowing plasma membrane anchoring (see reference [5]). As structure often defines function, the basic role of filamins is to organise actin filaments into orthogonal networks where the V-shape allows actin cross-linking in three-dimensions influencing cytoskeletal dynamics.

In addition to their basic function in actin dynamics, filamins serve as binding partners for a ∼90 proteins, scaffolding cell signalling platforms used to co-ordinate a myriad of cellular processes ranging from cell motility and signalling to organogenesis. In light of this, it is unsurprising that aberrant filamin action is implicated in a multitude of diseases including direct filaminopathies such as FLNA specific X-linked filaminopathies presenting with periventricular heterotropia (PH), a range of cancers (FLNA and B), dilated cardiomyopathy associated with a truncated FLNC-variant and thrombocytopenia (FLNA). Therefore, significant effort has been made to better understand the homeostatic regulation and pathogenic dysregulation of filamins. Imperative to this has been the quest to better appreciate the growing list of posttranslational modifications (PTMs) of filamins, with the most attention on filamin A. PTM of proteins is known to be diverse, ranging from reversable covalent addition of functional chemical groups to irreversible proteolytic cleavage, and this diversity allows spatiotemporal control over protein activity, location, dynamics and stability in a cell-type specific manner [6]. This article aims to provide an overview of the key PTMs of filamin A, with a particular focus on its phosphorylation, proteolytic cleavage and ubiquitin-proteasome mediated degradation. Advancements in scientific techniques has enhanced our understanding PTMs in the context of diseases and has potentiated development of novel therapeutic interventions that are focused on the PTM status of proteins, hence we also discuss the key PTMs of FLNA which may provide useful targets in disease.

## Phosphorylation

Phosphorylation is the covalent addition of a phosphate moiety to a free hydroxyl group located on the side chains of serine, threonine and tyrosine residues and is the most extensively studied protein post-translational modification with ∼75%–90% of the human proteome predicted to be amenable to phosphorylation [7]. As a key regulator of cytoskeletal organisation, cell migration, focal adhesion disassembly, cell membrane integrity and scaffolding of diverse signalling complexes, phosphorylation of filamin A at multiple residues co-ordinates its ligand interaction profile, actin binding status, expression, and susceptibility to cleavage [8]. Consequently, this modification is key for cell physiology and has the potential to be exploited as a target in a host of diseases including direct filaminopathies caused by X-linked filamin A variants, cancer, cardiovascular disease, and neurological disorders [9,10]. This review will cover some of the most important actions of filamin A phosphorylation.

## Filamin A in cytoskeletal remodelling, formation of focal adhesions and cell migration

### Phosphorylation by protein kinase A

Filamin A phosphorylation was first detected in human platelets where cAMP dependent protein kinase (protein kinase A/PKA) specific phosphorylation could be measured *in vitro* [11–14]. Filamin A has since shown to be phosphorylated in a variety of different cells including fibroblasts, smooth muscle, endothelial cells and human melanoma lines. There is robust evidence to suggest that filamin A is commonly modified by phosphorylation and that this ubiquitous PTM is essential for its function [11,15,16]. PKA phosphorylates a cadre of different proteins and is in itself highly regulated, both by inhibition of the active site via bound regulatory domains, which dissociate upon cAMP binding, but also spatially through scaffolding by A-kinase anchoring proteins forming discrete signalosomes allowing multiplexed responses to cAMP second messenger signalling [17]. PKA dependent phosphorylation of Serine2152 in the IgG20 repeat domain of filamin A was found to be protective against calpain mediated cleavage of filamin A, whereas phosphorylation at that site did not protect against trypsin, papain or thermolysin specific cleavage suggesting a calpain-specific mechanism [14,18,19]. This was replicated *in situ* where site directed mutagenesis confirmed the S2152 phosphorylation site and not T2336 [18].

The consequences of PKA modification of filamin A are tightly linked to the auto-inhibitory mechanism of Ig20 on Ig21. Ig20 occludes the Ig21 ligand docking site which binds proteins such as Integrin β-subunits and migfilin [19,20]. Filamin A interacts with the cytoplasmic domain of a range of integrin β-subunits (as well as binding to α-subunit N-terminal helices) directing the transmembrane receptor into a resting state, outcompeting β-subunit interaction with integrin activators such as talin [21,22]. As such, filamin A phosphorylation and consequent inactivation of integrins leads to a downstream reduction in integrin signalling which has profound effects on cell adhesion (through talins, vinculin and α-actinin), and acts to inhibit cell migration [22,23]. The Ig20 PKA consensus motif ^2148^RRAPSVA^2154^ is readily accessible and molecular modelling shows it neatly fits into the PKA active site, however, significant steric hindrance from Ig20 residues lock the complex into an auto-inhibitory conformation preventing S2152 phosphorylation. Only upon ligand binding of Ig21 to integrins and/or migfillin in the Ig19-24 repeat region, to relieve Ig20-Ig21 autoinhibition, does conformational change allow phosphorylation of S2152 [19]. Interestingly, in silico modelling suggests a bimodal mechanism to access the integrin binding site within filamin A Ig21 involving mechanical force and S2152 phosphorylation. This concept proposes a mechanosensory function of filamin A in anchoring F-actin networks to the cell membrane and matrix [24]. Models suggest that relief of Ig20-Ig21 auto-inhibition is maximised by dual effects of 40pN tensile force and S2152 phosphorylation, with phosphorylation alone not enough to allow integrin binding to Ig21. This data verifies that filamin A is essential in stimuli-specific cytoskeletal reorganisation through a force-phosphorylation dependent relationship [22,24].

The primary physiological consequences of filamin A phosphorylation by PKA are driven by changes in cytoskeletal organisation and in turn driven by filamin A cross-linking of actin filaments [8]. As phosphorylation is protective against cleavage by calpain, the immediate effects are not only elicited by modulation of filamin A interaction with integrins but also by F-actin's three-dimensional cytoskeletal organisation, which serves to strengthen actin-filament cross-linking into orthogonal networks or tight actin bundled stress fibres depending on the localised filamin:actin ratio. This modality is vital to the regulation of a plethora of key cellular processes [2].

PKA dependent phosphorylation of filamin A is required for normal endothelial barrier function [25,26]. The endothelial barrier is formed by tightly linked intercellular junctions between endothelial cells. These junctions compartmentalise the vascular and interstitial space allowing diffusive exchange of molecules. Pro-inflammatory agonists such as thrombin and bradykinin can increase vascular permeability by dismantling F-actin networks which form thick peripheral bands at endothelial junctions [2,3]. Activation of cell membrane adenylate cyclase 6 by Gα_s_ protein signalling in endothelial cells increases membrane-localised cAMP concentration near the surface membrane, in turn promoting PKA phosphorylation of filamin A S2152, which prevents cleavage by calpain and filamin translocation from caveolin-rich lipid raft domains to the cytosol [26,27]. Conversely alternate phosphorylation of filamin A by calcium-calmodulin kinase II (CaMKII) increases cytosolic translocation and disruption of the endothelial barrier by F-actin disassembly, via two distinct mechanisms. Firstly, bradykinin induces calcium (Ca^2+^) dependent activation CaMKII which phosphorylates the C-terminal region of filamin A (S2523) [28]. This action reduces filamin A actin-cross-linking ability and as such reduces endothelial barrier integrity [29,30]. Secondly, in response to ischaemia-reperfusion derived oxygen, free radicals and H_2_O_2_ cause a reduction in cAMP concentrations, impeding PKA S2152 filamin phosphorylation, resulting in impaired orthogonal F-actin cross-linking, increased paracellular gap formation leading to barrier disruption [25,30]. Thus, in endothelial cells studies there are polarised outcomes in endothelial barrier integrity as a result of differential kinase activity (namely PKA and CaMKII) on filamin A. The contrasting processes likely involve a multitude of different protein-interactions fine-tuned by filamin A phosphorylation, for instance one such interaction is with the R-Ras small G-protein at the plasma membrane, allowing VE-cadherin and c-Src, to modulate endothelial barrier leakiness, which has pertinent immunological consequences during bacterial invasion from blood to peripheral tissues [31].

Filamin A phosphorylation at S2152 can also be induced by direct binding to the C-terminal tail of activated GPCRs, allowing cytoskeletal remodelling by extracellular stimuli through a GPCR-filamin A-PKA dependent axis. Approximately 20% of the 824 GPCRs in the human proteome are predicted to contain a filamin A binding motif, predominantly contained in the C-terminal tail after intracellular loop 3 (ICL3), notably CCR2, D2 and D3 dopamine and calcitonin, μ-type opioid receptor, calcium-sensing receptor, somatostatin receptor type 2 receptors have been shown to interact with filamin A, but to date only the angiotensin type II receptor 1 (AT1R) has been characterised to induce filamin A phosphorylation after agonism with angiotensin II [20,32–39].

### Phosphorylation by p21 activated kinase and RSK in cell migration

Human melanoma cell lines lacking filamin A expression are known to be non-motile and display significant membrane blebbing. Interestingly, restoration of filamin A expression to WT levels rectifies the cell migratory properties. These data support the notion that filamin A represents an essential cog in the wheels driving cell locomotion [40]. Filamin A is also amenable to phosphorylation at S2152 by p21 activated kinase 1 (PAK1) where they both colocalise in membrane ruffles in the protruding membrane of motile cells [41]. PAK1 activity is regulated by small GTPases belonging to the Rho family, Rac1 and Cdc42, which co-ordinate cytoskeletal rearrangement in response to extracellular stimuli, such as growth factors like heregulin and sphingolipids. These can augment the filamin A-PAK1 interaction in MCF-7 breast cancer cells, with actin cytoskeleton integrity crucial to this interaction [41,42]. PAK1 binds filamin A Ig23 through its CRIB domain, relieving PAK1 autoinhibition in an identical manner to Rac1/Cdc42 PAK1 activation by T423 autophosphorylation [41]. Filamin A, PAK1 and sphingosine kinase 1 (SphK1) form a triad whose complex interactions with each other and various other protein partners are key to formation of lamellipodia formation at the leading edge of motile cells during taxis [43]. This triad forms a dynamic feed-forward mechanism in the formation and stabilisation of lamellipodia, where PAK1 binds and phosphorylates filamin A S2152, promoting direct filamin A - F-actin remodelling and also PAK1 activation and phosphorylation of its targets including LIM kinases, Arp2/3, cofilin and myosin light chain kinase to promote actin cytoskeleton contraction [35]. The role of filamin A in membrane ruffle and lamellipodia formation is therefore defined by its actin binding properties, its ability to rearrange three-dimensional actin networks as well as its capacity to initiate protein–protein interactions with signalling proteins such as PAK1 and SphK1.

**Figure 2.**
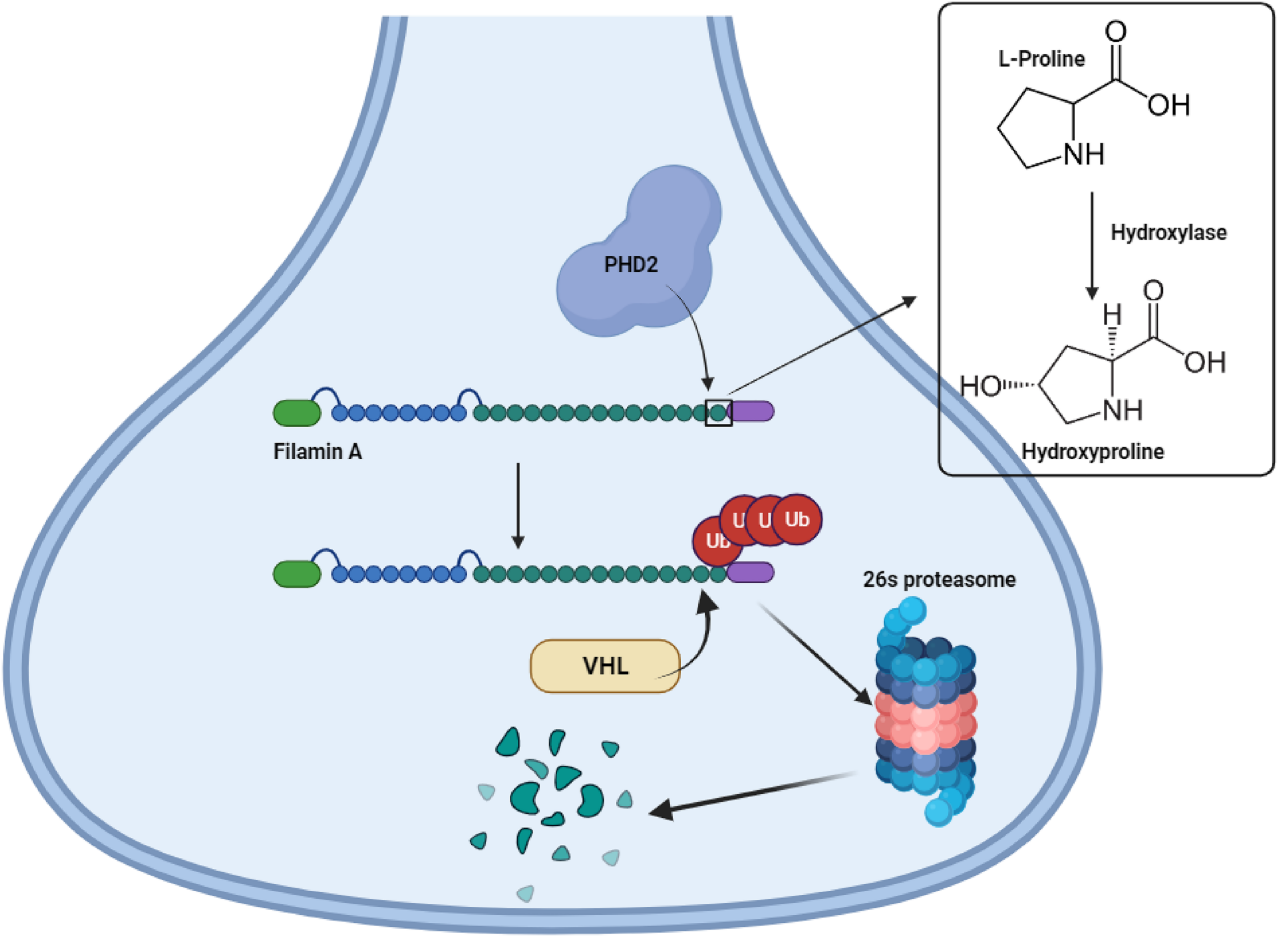
Filamin A hydroxylation by PHD2 in a neuronal dendritic spine. Filamin A is subject to hydroxylation at two proline residues: P2309 and P2316 by prolyl hydroxylase domain containing protein 2 (PHD2). Hydroxylation targets filamin A for polyubiquitination by Von Hippel Lindau E3 ligase (VHL) causing subsequent targeted protein degradation by the 26s proteasome. UPS mediated degradation of filamin A is essential for dendritic spine maturation from crude filopodia-like structures to mature spines capable of de novo synaptic transmission, vital for proper learning and memory [48]. Figure made using BioRender.

Filamin A is also a recognised substrate of p90 ribosomal protein S6 kinase (RSK), which phosphorylates at the promiscuous S2152 site [44,45]. RSK family kinases consist of RSK1-4, all containing two structurally differing kinase domains that are activated by multiple co-ordinated events such as phosphorylation by extracellular regulated kinase (ERK1/2), PDK1, autophosphorylation and/or subcellular localisation [44]. ERK1/2 phosphorylation is a consequence of the Ras-MAPK signalling cascade in response to growth factors such as epidermal growth factor (EGF) initiated by signalling though the membrane bound receptor tyrosine kinase EGFR [46]. Subcellular localisation of RSK is essential to its activation and thus function in phosphorylating a variety of transcriptional regulator substrates including CREB, NFκB, serum response factor and oestrogen receptor-α [47]. RSK proteins predominantly localise to the plasma membrane and nucleus where they co-localise with the upstream activator kinase ERK1/2, coordinating of cell motility in response to extracellular growth factors by translocation to the nucleus to regulate transcription [46]. The role of Ras-MAPK signalling in modulating cell cytoskeletal changes in relation to cell motility was previously ill defined, work by Woo et al. characterised filamin A as an essential factor in this through RSK selective phosphorylation of filamin A [44]. Constitutive targeting of RSK to the membrane causes increased phosphorylation of filamin A, augmenting filamin actin-binding and membrane cytoskeletal integrity. RSK translocation to the membrane to phosphorylate filamin A therefore represents a key mechanism in EGF-stimulated cytoskeleton remodelling through filamin A actin binding dynamics dependent on RSK phosphorylation (Figure 2) [45,46].

RSK phosphorylation of filamin A is necessary for efficient cell migration. Conclusive experimentation using the FLNa-deficient M2 human melanoma cell line showed that impaired migration of M2 cells could be rescued by transfection of WT filamin A but blocked again by treatment with the MEK inhibitor UO126 suggesting that MAPK controlled filamin A phosphorylation is essential in growth factor stimulated-cell migration [45,49]. Further investigation into potential mechanisms governing the effects of the MAPK-RSK-filamin A axis on cell migration in the A431 squamous carcinoma and DiFi colon cancer cell lines, characterised the pivotal role of α_5_β_1_ integrin in EGF stimulated cell migration [49]. Utilising an integrin dependent inside-out signalling mechanism, EGF stimulation of EGFR results in RSK phosphorylation of S2152 of filamin A relieving Ig20-21 autoinhibition and increasing ligand binding affinity of Ig21 to the cytoplasmic β_1_ integrin domain. This switches the fibronectin-binding α_5_β_1_ integrin to its inactive state, thereby reducing local cell adhesion and potentiating a migratory phenotype [19,21,49]. Recently a host of biochemical, cellular and structural biology techniques were developed to demonstrate competition binding of filamin A to the β_3_-domain of αIIbβ_3_ integrin with phosphatidylinositol-4,5 bisphosphate (PIP2) activated actin binding protein Talin in platelets [50]. Exquisite fluorescence resonance energy transfer studies showed that upon Talin activation by PIP2 and consequent displacement of filamin A from the αIIbβ3 integrin β3-domain, there is a spatiotemporal dependent reassociation of filamin A Ig21 to the αIIb domain of the now activated αIIbβ3 integrin, which in itself undergoes conformational change [50]. The mechanistic effects of this reassociation of filamin A to the active integrin-talin bound complex on the F-actin cytoskeleton mediated through both talin and filamin A are currently unexplored, however it is likely that filamin A phosphorylation has an integral role in this intricate switching mechanism between inside-out and outside-in integrin signalling that govern key processes such as focal adhesion, migration and cell homeostasis.

The importance of S2152 is supported by further evidence that three other kinases also phosphorylate filamin A at this site. The kinases involved are Akt (protein kinase B), Ndr2 and PKCα [51–54]. In the MCF-7 breast cancer cell, Akt was identified to phosphorylate filamin A at S2152 following insulin-like growth factor I stimulation, an effect abolished by inhibition with PI3K inhibitor wortmannin [42]. Interestingly, caveolin-1 can sequester filamin A and phospho-Akt into caveolae, discrete membrane domains rich in sphingolipids and cholesterol, to promote filamin-A phosphorylation at the peripheral membrane location to enhance cell migration in MCF-7 cells [42]. In a related process, arsenic induced carcinogenesis caused S2152A mutagenesis, nullified filamin-A S2152 phosphorylation by Akt and ameliorated cancer cell migration in the human bronchial epithelial cell line, BEAS-2B [55].

## Sites other than S2152 on filamin A are amenable to phosphorylation

As outlined in Table 1, several filamin A serine and tyrosine residues can be phosphorylated by a range of different kinases. Cyclin D1 and cyclin-dependent kinase 4 activity in G_1_-S-phase cell cycle transition is an important signalling axis in all cells, and increased Cyclin D1 expression/activity is associated with breast, colon and prostate cancers. Cyclin D1/Cdk4 specific phosphorylation of filamin A at S2152 and S1459 using mass spectrometry has been detected, suggesting that decreased filamin phosphorylation at S2152 and S1459 is associated with decreased wound healing-migration of MDA-MB-231 breast cancer cells [58]. During mitotic division cells adopt a more spherical shape to interphase counterparts, requiring extensive actin cytoskeleton remodelling with phosphorylation of filamin A residues S1084, S1436, S1459 and S1533 during mitosis by the Cyclin B1/Cdk1 heterodimer in HeLa and M2 cells shown to be critical for effective daughter cell separation during cytokinesis [81,82]. Filamin A is not exclusively phosphorylated at serine residues, with *in vitro* kinase assay data of p56^lck^, a lymphocyte specific src-family tyrosine kinase, phosphorylating tyrosine residues in a specific V8 peptide sequence of filamin A, in turn increasing actin network cross-linking as evidenced by dynamic light scattering experiments [59]. The function of tyrosine phosphorylation in Filamin A is yet to be further explored.

**Table 1.** The post translational modifications of Filamin A. Details of modification types, modification sites, enzymes involved and methodology used to investigate them

Modification	Residue/domain	Modifying enzyme	Methodology	Disease Relevance
Phosphorylation	S2152	PKA	*In vitro* kinase assay, site-directed mutagenesis [14,18]	Endothelial barrier dysfunction [30,31] Periventricular nodular heterotopias [56,57] Pituitary cancers [39] Prostate cancer [58] Breast cancer [58] Squamous cell carcinoma [49] Colon cancer [49]
	S2152	PAK1	*In vitro* kinase assay [41]	
	S2152	AKT	Mass spectrometry (MS), *in vivo* IGF-I stimulation + wortmannin PI3K inhibitor immunoblotting [51]	
	S2523	CamKII	*In vitro* kinase assay [29]	
	S2152	RSK	Peptide kinase assay, *in vivo* EGF stimulation + UO126 treatment immunoblotting [44,45]	
	S2152	PKC-α	PKC inhibitor treatment + immunoblotting, cell 32-orthophosphate labelling + immunoblotting, site directed mutagenesis + immunoblotting [53,54]	
	S2152	Ndr2	*In vitro* kinase assay, peptide array [52]	
	S1459 and S2152	Cyclin D1/Cdk4	MALDi-MS, Western blotting [58]	
	Tyrosine residue(s) in a V8 peptide sequence	p56lck	Co-immunopurification, *in vitro* kinase assay [59]	
Cleavage	Hinge 1 between Ig15-16 resulting in two fragments: 170 and 110 kDa	Calpain 1/2	*In vitro* Calpain incubation and immunoblotting [28]	Prostate cancer [60–65] Melanoma [66,67] Mitral valve dystrophy [68,69] Aortic aneurysm in Marfan's syndrome [70] Atherosclerosis [71,72]
	Hinge 2 cleavage of 110 kDa fragment between Ig-23-24 resulting in a 90 kDa C-terminal fragment	Calpain 1/2	Western blotting prostate cancer cell lysate [73]	
Ubiquitination	K42, K43, K135 CH1 domain	Asb2α	Immunofluorescence, MG132 treatment and western blotting [74]	Acute myeloid leukaemia (AML) [74,75] Tuberous sclerosis and neuronal development [72,76,77]
	Ig21-23	Von Hippel Lindau (VHL)	HEK293T cell MG132 treatment, western blotting and mutagenesis [77]	
Hydroxylation	P2309 and P2316	PHD2	Hydroxy-proline specific monoclonal antibody + MG132 treatment and LC–MS [77]	Tuberous sclerosis and neuronal development [72,76,77]
O-GlcNAcylation	Unknown	O-GlcNAc transferase (OGT)	BioID proximity biotinylation plus stable isotopic labelling of amino acids in cell culture (SILAC) and co-immunoprecipitation [78]	Unexplored to date but implicated in oxidative stress [78]
Acetylation	Five lysine residues	P300 KAC	Proteomics based approach [79]	Thrombosis and clotting disorders [79]
Carbonylation	Unknown	Chemically driven reaction	ESI-LC–MS/MS from obese mice adipose [80]	Obesity induced insulin resistance

## Filamin A dephosphorylation

As phosphorylation is a dynamic and reversible PTM, dephosphorylation is every bit as important as dephosphorylation and filamin A is not exempt from this paradigm. There are however significantly fewer phosphatases than kinases, with ∼200 phosphatases in the human proteome [83]. In platelets, calcineurin, a ser/thr specific-phosphatase activated by calmodulin binding and intracellular [Ca^2+^] increase, was found to dephosphorylate the PKA specific phosphorylation of S2152 in the filamin A C-terminal region. Functionally, dephosphorylation increased the proteolytic cleavage of filamin by calpain, where protection is usually conferred through S2152 phosphorylation [84]. Cyclosporin, a known inhibitor of calcineurin, was able to rescue filamin A from calpain cleavage representing a method to study the effects on downstream signalling of filamin derived peptide fragments [47]. Finally, it is known that protein phosphatase 2A (PP2A) was able to remove CaMKII specific S2152 phosphorylation *in vitro* but more research is required to better understand the *in vivo* mechanisms of filamin A dephosphorylation in order to exploit any therapeutic benefits [29,44].

## Regulation of filamin A phosphorylation status in disease

Filamin-A is implicated in a host of different diseases and its phosphorylation state is germane to some of them. For instance, studies investigating causes of PH, an X-linked developmental condition causing congenital cortical malformation, found increased S2152 phosphorylation by PKA disrupted neuronal progenitor migration from the ventricular zone to the cortical plate during brain development. This defect was mediated through the action of ADP-ribosylation factor guanine exchange factor 2 gene (ARFGEF2) encoding brefeldin A-inhibited guanine exchange factor 2 (Big2) protein [56,57]. Due to its significant roles in cytoskeletal remodelling affecting cell adhesion, migration, mitosis and signalling, filamin A is implicated in cancer pathophysiology, and particularly tumour cell metastasis. Here, filamin A switches between tumour suppressive or oncogenic roles dependent on the specific cancer as well as cellular localisation of filamin A [85]. For example, in growth-hormone secreting pituitary tumour cells, cAMP-dependent phosphorylation of filamin A inhibits signalling of the Gi linked GPCR SS-receptor type 2 (SST2). In this case, S2152 phosphorylation negatively regulates SST2 anti-tumoral signalling and reduces sensitivity to somatostatin analogues used to treat pituitary cancer. Hence targeting this site represents a potential avenue to rescue tumoral resistance to SSAs [39,86]. However, targeting filamin A phosphorylation itself is a difficult task. Selective inhibitors of specific kinases or phosphatases may offer depletion and/or promotion of filamin phosphorylation, however diligence would be needed in monitoring off-target effects due to filamin A's pivotal role in organogenesis, and cell homeostasis. Current advancements which could pave the way in selective-targeting of filamin A phosphorylation include development of heterobifunctional molecules capable of recruiting specific kinases or phosphatases to induce (de)phosphorylation of a defined target [87]. Difficulties in targeting filamin A with such modalities derive from the Ig-repeats which may interfere with specificity in targeting a single serine residue such as S2152 with a heterobifunctional molecule. Small peptide disruptors mimicking the specific amino-acid sequence of the filamin-A kinase interaction interface could potentially be beneficial in this instance, allowing targeted disruption of the protein-protein interaction preventing phosphorylation or dephosphorylation [88].

Overall, filamin A is known to be phosphorylated at a range of serine residues (predominantly at S2152) to modulate functions in cell migration, focal adhesion, signalling and mitosis. This involves a host of different kinases reflecting the signal and cell-specific mechanisms inducing filamin A phosphorylation. Better characterisation of the ∼28 predicted phosphosites is required, for instance tyrosine phosphorylation sites have been shown to be detected in vitro, and narrowed to a single V8 peptide, but further functional studies in a different cell-lines are needed to investigate the relevance of phosphorylation in vivo [89]. Given the multi-faceted roles of filamin A, targeting its phosphorylation in different disease may offer potential in alleviating disease phenotypes, for instance in metastatic progression of breast and prostate cancers, compared with a traditional pharmacological inhibition approach which may be unfavourable given filamin A's complex scaffolding of orthogonal actin networks as wells as discrete signalosomes vital for compartmentalised responses to different stimuli. As the primary elucidated role of FLNA phosphorylation is to prevent calpain dependent cleavage, and as phosphorylation-dephosphorylation cycling of filamin A likely exists in a dynamic state dependent on other unknown, this would in theory make targeting phosphorylation of filamin A therapeutically difficult. More ground-work is needed to determine if multiple-kinase mediated phosphorylation of filamin A is specific to; cell-type, the stage of the cell-cycle, specific cellular compartments, and/or only evident upon onset of specific disease changes. This may prompt development of disease specific therapeutic strategies targeting filamin A phosphorylation.

## Cleavage

In addition to phosphorylation, filamin A is regulated by the irreversible post-translational modification, proteolytic cleavage, which is an enzymatic process catalysed by many different proteases (e.g. calpain, caspases) [5]. Each protease recognises a different sequence of amino acids on filamin A that allows recruitment of the protease and subsequent proteolysis at the motif [90]. Proteolytic cleavage of filamin A is induced by a variety of cues with different fragments of filamin A being produced in a stimuli-dependent fashion to illicit defined cellular outcomes. filamin A is cleaved by calpain on two hinge regions [28,91]. The first region is situated between repeats 15 and 16. Following cleavage at this site, two fragments are generated a 170 kDa actin-binding N-terminal fragment, which terminates at repeat 15 and a smaller 110 kDa C-terminal fragment (between repeats 16–24). The larger N-terminal fragment retains the actin-binding domain, and some motifs responsible for protein-protein interactions and cytoskeletal organisation. The C-terminal fragment contains the dimerisation domain, essential for forming filamin A dimers and encompasses the second half of the rod domain [4,5]. There are distinct variations between the binding affinity for some partner molecules of the whole filamin A molecule compared with the one of the fragments. For instance, some of the amino acid sequences that are present in the intact molecule could either hinder or promote a certain process if removed in a splice variant. One such example is related to a splice variant of filamin A, lacking a 41-amino acid segment, and its binding ability to the integrin b7 cytoplasmic domain [92]. It has been proven that the fragment binds more strongly to the integrin in comparison with the whole filamin A protein. Phosphorylation mediated decrease in affinity for the intact fragment is unlikely, because the major PKA phosphorylation site within the molecule is between the 19–24 repeats. This repeat is present in the splice variant as well; but it is possible that other distant phosphorylation sites could negatively influence the binding of whole filamin A with integrin — a concept which would need experimental validation. This is a vivid example of the diverse and sometimes opposite properties of filamin A and its cleaved fragments.

The consequences of filamin A cleavage are diverse and affect important cell processes such as cytoskeletal organisation and cell migration through loss of filamin A function. Altered cellular homeostasis is also driven by the action of different filamin A fragments which promote specific signal transduction alterations depending on their size and sequence. Needless to say, as the fragmentation of filamin A by proteolytic cleavage is irreversible, it is associated with many diseases including cancer and disorders of the cardiovascular and nervous systems [93].

## Filamin A cleavage and cardiovascular disease

Filamin A is cleavage is associated with degeneration of cardiac valves [68,69]. Specifically, mutations that alter the spatial and temporal expression pattern of filamin A during heart valve morphogenesis can result in cardiac valvular dystrophy [94]. Mutations in filamin A are also directly implicated in aortic aneurysms in patients with PH. Patients with the heritable connective tissue disorder Marfan syndrome (MFS) present with dilated aortic media and bicuspid aortic valve which predisposes aortic dissection, thoracic aortic aneurysm and premature death due to mutation in the fibrillin-1 gene [95]. Mass spectrometric studies in MFS patient aortic samples exhibited elevated levels of the C-terminal fragment of filamin A suggesting increased filamin cleavage [70]. The activity of calpain 2 within the cell is controlled temporally and spatially by factors such as Ca^2+^ concentration, the enzyme's cellular location and expression of its endogenously expressed inhibitor protein, calpastatin. In the case of MFS, increased calpain 2 expression and decreased calpastatin levels, drive excess formation of the C-terminal fragment [9]. Filamin A possesses the ability to co-translocate to the nucleus with the androgen receptor (AR), repressing its transcriptional activity [96,97]. Interestingly it has been reported in murine models that increased androgen levels and thus AR activity and exacerbate aortic dissection by enhancing aneurysm growth in a male *Fbn1*^C1039G/+^ mouse model [98]. More investigation is required to unpick a potential role of a filamin A C-terminal fragment- AR axis in MFS, with utility of *in vitro* and *in vivo* models of MFS imperative for unlocking therapeutic potential.

The C-terminal fragment of filamin A is also implicated in atherosclerosis. In this context, attenuation of filamin A cleavage following pharmacological inhibition of calpain impaired macrophage signalling and reduced atherosclerosis in mouse models where atherogenesis had been induced by hypercholesterolaemia [71]. Mechanistically, protection against atherosclerosis was driven by a paucity of the C-terminal fragment which binds to the transcription factor phospho-STAT3 to aid nuclear translocation and enable transcription of cytokines such as IL-6. Interestingly, the ability of the C-terminal fragment to recruit transcription factors has also been crucial in the cellular response to hypoxia whereby it binds to HIF-1a, enhances its nuclear translocation and transactivation function enabling expression of proteins such as GLUT1 [72].

## Filamin A cleavage and cancer

Several studies have evidenced that filamin A cleavage is a double-edged sword in the promotion or repression of cancer metastasis in a tumour tissue-dependent context [60]. In prostate cancer filamin A is speculated to possess tumour-supressing properties [61]. The AR (AR) was one of the first transcriptional regulators shown to directly associate with filamin A, indeed nuclear translocation of AR was blocked in filamin deficient cells even after treatment with synthetic ligand mibolerone [62,73,97]. Subsequent work specifically identified the filamin A C-terminal fragment as a direct binder of a motif on the AR within its negative regulatory hinge region [96]. Association of filamin A with the AR represses AR transactivation by competing with co-activator proteins and this was one of the first indications that filamin A was not only a structural protein but also a transcriptional regulator. As increased activity AR is heavily implicated in prostate cancer, the characterisation of filamin A's role in this disease has received much attention.

Firstly, filamin A has been identified as a translocator of prostate-specific membrane antigen (PMSA) protein which binds to the cytoplasmic tail of the integral membrane protein. PMSA is a highly characterised biomarker for prostate cancer as it is overexpressed in the solid tumour compared with the vasculature of healthy tissue [99]. Filamin A maintains the membrane localisation of PMSA by inhibiting its removal from the surface by recycling endosomes and hence plays a part in the detectability of this diagnostic protein [100].

Further to its role in prostate cancer in directly binding the AR and maintenance of membrane localised PMSA filamin A cleavage by calpain represses tumour metastasis in AR-positive prostate cancer, and its subcellular localisation is key to this [62,73]. Filamin A localisation depends on the type and progression of prostate cancer. In the case of AR positive prostate cancer cytoplasmic filamin A is cleaved and the 90 kDa fragment translocates to the nucleus, where it inhibits AR activity, resulting in decreased metastatic potential. Phosphorylation of filamin A at S2152 prevents calpain mediated cleavage, and as such prevents translocation of the 90 kDa fragment to the nucleus, averting direct fragment binding and inhibition of nuclear-localised AR. Promoting filamin A cleavage in androgen-dependent prostate cancer may therefore hold therapeutic advantage in preventing tumour metastasis, by increasing cleaved filamin A product nuclear translocation and inhibition of AR. Such potential therapeutics include Genistein combined polysaccharide, which is a natural product, promotes filamin A nuclear localisation by cleavage, and is a recognised as a potential treatment of prostate cancer [63].

Therapies modulating the filamin A/AR axis will require high specificity targeting. Recently the role of the filamin A/AR signalling axis was uncovered in prostate cancer associated fibroblasts (pCAFs) [101]. pCAFs are recruited by cancer cells to remodel the tumour microenvironment to enhance tumour growth and metastasis. pCAFs express an appreciable amount of AR with no transcriptional activity, and after androgen challenge association with the non-cleaved filamin A dimer triggers assembly of a membrane bound filamin A/AR/β1 integrin/MT-matrix metalloproteinase 1 (MT-MMP1) complex. MT-MMP1 activation in the extracellular space allows cleavage of the inactive pro-MMP-2 protein to active MMP-2, resulting in increased extracellular matrix degradation and augmented pCAF invasion into the prostate tumour. Preventing filamin A/AR complex assembly in pCAFs using a stapled peptide resulted in reduction in size of three-dimensional LNCaP/pCAF cell spheroids hence targeting this axis with highly specific peptide-disruptors may serve to treat prostate tumour growth and metastasis or alternatively as a novel biomarker [101].

Targeting filamin A phosphorylation is another viable option to impede prostate tumour metastasis. Pharmacological inhibition of one putative filamin kinase PKA with small molecules such as H-89 would prevent phosphorylation of filamin A, allowing cleavage by calpain and the aforementioned anti-metastatic effects. However, diverse and complex signalling networks control filamin A phosphorylation, with numerous kinases capable of S2152 specific phosphorylation, leaving potential for compensatory mechanisms upon inhibition of PKA. Contradictory to its role in AR-positive prostate cancer, filamin A cleavage by calpain augments tumour cell metastasis in a calcium-dependent manner. Calcium influx was found to increase cell migration in AR-deficient metastatic cell lines DU145 and PC-3 calpain cleavage of filamin A in AR-deficient metastatic prostate cancer cell lines [64].This is most likely due to the cellular redistribution of calpain, induced by calcium (Ca^2+^). Calcium influx was shown to modulate metastatic migration through a calcium sensing receptor (CaR)- p115RhoGEF-calpain signalling axis, culminating in filamin A cleavage. This situation, however, is not replicated in the androgen sensitive prostate cancer cell line LNCaP. In contrast, in castration resistant prostate cancer filamin A is largely confined to the cytoplasm which is associated with increased metastatic potential and a hormone-refractory phenotype [64]. Decreased nuclear and elevated cytoplasmic localisation of filamin A is apparent in the androgen-refractory (hormone-resistant) prostate cancer cells. Evidenced by immunohistochemical and *in vitro* studies, prostate cancer cell invasion is promoted by cytoplasmic localisation of filamin A and suppressed by filamin nuclear localisation [64].

In other cancers such as lung, breast, squamous cell carcinoma, fibrosarcoma and glioma, cleavage and nuclear translocation of filamin A promotes metastatic transition with the propensity of filamin A to bind to growth factors and regulate their expression being key to this. For instance, HIF-1α transcription factor in cancerous cells binds the 90 kDa cleaved filamin fragment, before co-translocation to the nucleus [65]. Thus, cleavage of filamin A propagates the promotion or repression of tumour metastasis, in context-dependent manner, at the tissue level determined by the stage of cancer progression and at the molecular level influenced by the binding partners of filamin A and as such activation of differential signalling pathways.

Filamin A cleavage plays a pivotal role in melanoma cells, particularly during metastasis [66]. Wnt5A signalling was found to directly augment motility of human melanoma cells, with further investigations characterising a putative Wnt5a-ROR2-Calpain axis (ROR2 is an orphan tyrosine kinase receptor previously shown to interact with Wnt5a) culminating in filamin cleavage, cytoskeletal remodelling and increased metastatic potential [67]. Wnt5A signalling through ROR2 activates an increase in intracellular calcium and induces filamin A cleavage through subsequent activation of calpain 1. This Wnt5A/ROR2 signalling cascade promotes filamin cleavage by calpain 1 which in turn leads to increased melanoma cell motility. This insight may offer a new therapeutic avenue by direct inhibition of Wnt5a or calpain 1 to prevent filamin cleavage, as well as the potential for increased cleaved filamin A product and/or Wnt5a serving as a useful metastatic biomarker.

## Ubiquitination

Filamin A concentration in the cell cytoplasm is additionally controlled through targeted degradation by the ubiquitin-proteasome system (UPS), adding another layer of complexity in organisation of actin-networking by filamin A. Filamin A and B isoforms are polyubiquitinated by the E3-ligase Asb2α at the N-terminal actin binding domain, where Asb2α serves as the specificity subunit of a CUL5-family RING E3 complex [74,102,103]. Neat biochemical studies identified three lysine residues (K42, K43 and K135) in the actin binding calponin-homology 1 domain as the targets of both Asb2α binding and covalent linkage of ubiquitin, indeed augmenting expression of Asb2α in NIH3T3 cells reduced cell spreading, likely due to subsequent degradation of filamin A and B [74]. Physiologically filamin A and B degradation is key to haematopoietic stem cell differentiation, thought to be mediated through modulation of cell spreading and actin remodelling. Pathophysiologically this is a key determinant in progression of acute myeloid leukaemia (AML), where haematopoietic stem cells arrest at an immature stage of cell differentiation, resulting in accumulation of immature cells in the bone marrow and blood [74,102]. Asb2α was identified as an important regulator in the therapeutic effect of all-trans retinoic acid (ATRA) in AML, where ATRA acts on acute promyelocytic cells (APL) causing stem cell maturation [75]. Critically, activation of Asb2α in APL cells causes ubiquitin mediated degradation of filamin-A, suggesting this mechanism is key to APL differentiation through cytoskeletal remodelling seen in the reduced spreading phenotype [75].

Filamin A ubiquitination is additionally key to Natural Killer (NK) cell migration to the tumour microenvironment, controlled by the aryl-hydrocarbon receptor (AHR). NK cells belong to the innate immune system and their primary role is exerted through blood and tissue infiltration, targeting and destroying viral infected or tumour cells. The AHR is a ligand binding transcription factor essential for effective NK cell migration into the tumour microenvironment, part of a network of receptors key to NK cell migration (e.g. chemokine receptors) [104]. The mechanism behind AHR-deficiency in impeding migration of NK cells to tumours was deciphered through a AHR^−^/^−^ knockout cell-line [48]. Asb2α mRNA and protein expression was decreased in AHR^−^/^−^ NK-cells, and further investigation yielded that AHR activation by its cognate ligand FICZ caused filamin-A degradation through Asb2α specific ubiquitination [48]. This exemplifies the necessity of filamin-A ubiquitination in linking extracellular stimuli and provocation of a specific cell-response primarily by re-organisation of the actin cytoskeletal network. The wider context of filamin A ubiquitination in NK-cells in treatment of malignant tumours is currently unexplored, and further investigations may yield promising results, deepened further by current excitement surrounding immunotherapy in treating cancer.

## Filamin A tyrosylation, hydroxylation and O-GlcNAcylation and more

As the advances in modern techniques has rapidly expanded our appreciation of the myriad of possible PTM types [6] it is likely that we have only begun to chart the modification landscape of filamin A. Tyrosylation involves the modification of residues under oxidative stress conditions generating tyrosyl free radicals as a result of reactive oxygen species hydrogen peroxide (H_2_O_2_) reacting with L-tyrosine, these radicals react with and bond to a range of amino acid side chains including, tyrosine, glutamic acid and lysine [105–107]. During activation of neutrophils in the anti-inflammatory response many cytoskeletal proteins are tyrosylated including filamin A and vimentin. The functional importance of such modifications is currently unknown and warrants further investigation [64].

Filamin A is essential in synaptic plasticity through its roles in dendrite morphogenesis, cytoskeletal remodelling during neuronal migration and axonal growth cone formation [72,76,108]. Synaptic transmission of action potentials is an energy consuming process reliant on high ATP generation primarily through oxygen-dependent oxidative phosphorylation, this metabolic demand is impaired during hypoxia and thus impedes efficiency of synaptic transmission. Under normoxic conditions the cellular oxygen sensors, prolyl hydroxylase domain containing proteins (PHDs), inhibit hypoxia-inducible factor 1α (HIF1α) activity via proline residue hydroxylation and subsequent UPS degradation mediated by E3 ligase Von Hippel-Lindau (VHL). HIF1α is an important transcription factor controlling expression of genes related to angiogenesis, proliferation and glucose/iron metabolism to better cell survival during hypoxia, hydroxylation and subsequent UPS mediated degradation silences HIF1α activity acting as an important mechanism to direct cell homeostasis under normoxic conditions [109]. Hypoxic conditions in the brain cause reduced action potential firing, detraction of dendritic spines, and reduced synaptic density and recently a filamin A/PHD2 signalling axis was elucidated to be involved in this [77]. Dendritic spines are small membrane protrusions at the neuronal dendrite surface which serve to extend and develop de novo synapses by receiving nerve impulses from presynaptic terminal of proximal neurons, hence are functionally important in learning and memory [110]. During normoxia filamin A proline residues P2309 and P2316 are hydroxylated by PHD2 through the addition of a hydroxyl -OH group to Carbon-4 of the residue, targeting filamin A for UPS mediated degradation by VHL E3 ligase. This promotes mature dendritic spine formation from an initial filopodia like structure to a recognisable structure protruding from the main dendrite body composed of a thin spine neck extending to a spine head. Silencing PHD2 activity and up-regulating filamin A causes immature spine formation phenocopying the hypoxic response in the brain [48]. In the wider context it is still not understood how up-regulation of filamin A causes immature spine formation as both the overexpression and knockout of filamin A in neurons impairs neuronal migration and proper spine homeostasis suggesting the requirement for filamin A to be under tight regulation in neurons [105]. Filamin A beyond its role as a key component in cytoskeletal remodelling interacts with numerous proteins, which may in part explain how it's over or under expression exhibit similar effects on spine formation. Potential signalling proteins filamin A may associate with in dendritic spines are small GTPases Rac and Cdc42 which have been shown to promote mature spine formation however this requires experimental confirmation.

Filamin A will undoubtedly be amenable to other modification types with various proteomic based experiments identifying potential; O-GlcNAcylation, carbonylation and acetylation sites [78–80]. O-GlcNAcylation is the addition of the *O-*linked-*N*-acetylglucosamine monosaccharide to serine/threonine residues catalysed by the enzyme O-GlcNAc transferase (OGT), and removed by O-GlcNAcase enzyme (OGA) [111]. Utilising proximity-biotinylation to probe for protein-protein interactions coupled with stable isotope labelling of amino acids in culture (SILAC)-mass spectrometry, OGA was found to be associated with 90 proteins including filamin A and fatty acid synthase and these modifications were further validated by co-immunoprecipitation. While it is hypothesised that filamin A O-GlcNAcylation is low under basal levels, it will be induced under induction of oxidative stress [105]. While this is not direct evidence of the importance or relevance of filamin A O-GlcNAcylation functionally, it does open the potential for investigation of specific filamin A O-GlcNAcylation in apt rodent models of cardiac ischaemia reperfusion injury [107]. In the context of filamin A, O-GlcNAcylation may influence cytoskeletal responses to cell stress through the various filamin A-cytoskeletal interactions, however this needs significant validation through biochemical and physiological studies. There is also preliminary evidence of filamin A carbonylation, shown in adipose proteins isolated diet induced obese C57Bl/6J mice, filamin A was identified to be post-translationally modified by addition of *trans*-4-hydroxy-2-nonenal (4-HNE), a reactive aldehyde a by-product of reactive oxygen species mediated lipid peroxidation [80] Significantly more investigation into filamin A carbonylation is required to underpin any functional meaning to this modification. Notably oxidative stress caused by inflammation or obesity induced insulin resistance causes an increase in the pool of 4-HNE and other reactive aldehydes which are not metabolised or conjugated to glutathione, resulting in a global increase in protein carbonylation, potentially only eliciting functional effects under these specific conditions. As filamin A has been shown to interact with the insulin receptor to inhibit its function, filamin A modification with 4-HNE may be relevant in adipocyte insulin resistance mechanisms [80].

Filamin A is vital for platelet function following GPVI activation by C-reactive protein and the role of acetylation in modulation of platelet cytoskeletal function has been investigated [112]. Filamin A was found to be acetylated at ∼5 lysine sites by the Src-kinase activated lysine acetyltransferase p300 enzyme (p300 KAC) [79]. P300 KAC inhibition resulted in reduced acetylation of actin, filamin and cortactin. This resulted in measurable cytoskeletal effects in the context of filamin A, integrin α_IIb_β_3_ activation and aggregation and is in line with previous evidence linking filamin mediated integrin inactivation potentiating filamin-A acetylation as a mechanism of negatively modulating the filamin A-integrin interaction. Importantly blocking p300 KAC activity in platelets reduced aggregation under shear stress, giving it physiological relevance.

## Conclusion

In summary, our review provides an up-to-date picture of the well characterised and newly identified PTMs of Filamin A. Clearly the broad range of Filamin A's functional capabilities is enriched in cells by these modifications and fine tuning Filamin A's coordination of cellular signalling and physiological processes can be reckoned using these chemical additions and cleavage events. There is also an obvious link to a variety of diseases where the aberrant PTM of Filamin A plays a role. Identification of new Filamin A PTMs using modern ‘omics’ technology is inevitable and these along with the ones captured here may provide novel avenues for the development of innovative therapeutic strategies.

## Abbreviations

AML: acute myeloid leukaemia
AR: androgen receptor
AT1R: angiotensin type II receptor 1
CaMKII: calcium-calmodulin kinase II
Ca^2+^: calcium
ERK1/2: extracellular regulated kinase
EGF: epidermal growth factor
FLNA: filamin A
FLNC: filamin C
ICL3: intracellular loop 3
MFS: Marfan syndrome
MTMMP1: MT-matrix metalloproteinase 1
PAK1: p21 activated kinase 1
PCAF: prostate cancer associated fibroblast
PH: periventricular heterotropia
PKA: cAMP dependent protein kinase
PKC: protein kinase C
PMSA: prostate-specific membrane antigen
PP2S: phosphatase 2A (PP2A)
PTM: post translational modification
RSK: ribosomal protein S6 kinase
Sphk1: sphingosine kinase 1
UPS: ubiquitin-proteosome system
VHL: Von Hippel-Lindau
